# Degradability of Biodegradable Soil Moisture Sensor Components and Their Effect on Maize (*Zea mays* L.) Growth

**DOI:** 10.3390/s20216154

**Published:** 2020-10-29

**Authors:** Subash Dahal, Wubengeda Yilma, Yongkun Sui, Madhur Atreya, Samantha Bryan, Valerie Davis, Gregory Lewis Whiting, Raj Khosla

**Affiliations:** 1 Department of Soil and Crop Sciences, Colorado State University, Fort Collins, CO 80523-1170, USA; subash.dahal@colostate.edu (S.D.); wub.yilma@colostate.edu (W.Y.); samisam@rams.colostate.edu (S.B.); valerie.davis@colostate.edu (V.D.); 2 Paul M. Rady Department of Mechanical Engineering, University of Colorado Boulder, Boulder, CO 80309-0427, USA; yongkun.sui@colorado.edu (Y.S.); madhur.atreya@colorado.edu (M.A.); gregory.whiting@colorado.edu (G.L.W.)

**Keywords:** soil moisture sensors, degradability, maize growth and development

## Abstract

Inexpensive and no-maintenance biodegradable soil moisture sensors could improve existing knowledge on spatial and temporal variability of available soil water at field-scale. Such sensors can unlock the full potential of variable-rate irrigation (VRI) systems to optimize water applications in irrigated cropping systems. The objectives of this study were to assess (i) the degradation of soil moisture sensor component materials and (ii) the effects of material degradation on maize (*Zea Mays* L.) growth and development. This study was conducted in a greenhouse at Colorado State University, Colorado, USA, by planting maize seeds in pots filled with three growing media (field soil, silica sand, and Promix commercial potting media). The degradation rate of five candidate sensor materials (three blends of beeswax and soy wax, balsa wood, and PHBV (poly(3-hydroxybutyrate-co-3-hydroxyvalerate))) was assessed by harvesting sensor materials at four maize growth stages (30, 60, 90, and 120 days after transplanting). All materials under consideration showed stability in terms of mass and dimension except PHBV. PHBV was degraded entirely within 30 days in soil and Promix, and within 60 days in sand. Balsa wood did now show any significant reduction in mass and dimensions in all growth media. Similarly, there was no significant mass loss across wax blends (*p* = 0.05) at any growth stage, with a few exceptions. Among the wax blends, 3:1 (beeswax:soy wax) was the most stable blend in terms of mass and dimension with no surface cracks, making it a suitable encapsulant for soil sensor. All materials under consideration did not have any significant effect on maize growth (dry biomass, green biomass, and height) as compared to control plants. These results indicated that 3:1 beeswax:soy wax blend, PHBV, and balsa wood could be suitable candidates for various components of biodegradable soil moisture sensors.

## 1. Introduction

Variable-rate irrigation (VRI) is a tool of precision agriculture that is aimed at optimizing water use by applying the right amount of water, at the right place, at the right time, in the right manner. Crop producers already possess the VRI technology that can control water application at a fine spatial and temporal resolution. However, producers lack the technology to measure plant-available soil water at a spatial and temporal resolution that is required to fully harness the capability of VRI systems to optimize water use. A major roadblock in developing a highly optimized variable rate irrigation system is the lack of ability to measure soil moisture at spatially and temporally dependent scales.

We have come a long way in the field of soil moisture measurement from direct gravimetric measurement to the use of various sensor-based measurements, such as neutron probes [1,2], microwave sensors [3], dielectric techniques [4], optical sensors [5], thermal sensors [6], and tensiometric sensors [7]. With technological advancements, moisture sensors are becoming smaller, cheaper, and more reliable; however, placement of such sensors in the field at high density to collect data at a spatially dependent scale (i) is cost-prohibitive, (ii) results in significant electrical and plastic waste in soil, and (iii) requires labor-intensive retrieval and maintenance, rendering them unsuitable for high resolution soil moisture data collection.

A possible solution to resolve the abovementioned issues of current soil moisture sensors is to develop cheap sensors from biodegradable components that could eliminate electrical and plastic waste, maintenance, and retrieval requirements, as well as provide reliable soil moisture data at high spatial resolution. However, developing biodegradable soil sensors is easier said than done. There are numerous concerns that need to be addressed, such as (i) selection and screening of suitable materials, (ii) drift in measurement due to degradable components, and (iii) effect of component materials in crop growth and development. Recently, patents have been filed [8,9], indicating a possibility of developing biodegradable sensors by using biodegradable materials apart from a conductive bioinert circuit and antenna.

Although biodegradable sensors have long been studied and used in other disciplines such as medicine [10,11,12,13,14] and engineering [15,16], few efforts [17] have been made to develop sensors that are suitable for agricultural use. Jiang et al. [17] utilized a biodegradable encapsulation material coated in different thickness onto bioinert electrodes to time the degradation so that a series of identical electrodes can be sequentially exposed to soil to extend the functional lifetime of the sensor. We propose a capacitive soil moisture sensor with a hydrophobic encapsulant, which will sense the dielectric constant change of the surroundings and will read by a radio frequency identification (RFID) reader. A thin layer of the encapsulant will not completely block the electric field generated by the electrodes; thus, the dielectric constant change of the soil due to water content variation can still be measured. We choose a hydrophobic material as the encapsulant because we intend to use biodegradable materials as the electrical conductor for the moisture sensor (e.g., zinc) to further reduce the impact to the environment. The slow-degrading hydrophobic encapsulant can protect the biodegradable conductor from premature degradation. Ideally, the sensor will be buried deep in the soil; the antenna, however, will stay above ground to reduce loss in signal strength. The sensor and the antenna will be electrically connected using a conductor which will be done by using wax-encapsulated zinc. The results of this preliminary study could be a stepping stone for producing low-cost, maintenance-free, biodegradable soil sensors for use in precision agriculture. The main advantage of having biodegradable sensors is that there will be no maintenance cost because the sensors will degrade after the cropping season. Moreover, the sensors will be developed in such a way that there is no need for batteries and other electrical waste. Being extremely small (few centimeters), inexpensive (<$1), and without maintenance and retrieval requirement, these sensors could revolutionize the way we farm and irrigate.

Poly(3-hydroxybutyrate-co-3-hydroxyvalerate) (PHBV) and its derivatives have long been used in the medical field as encapsulants of drugs for delayed-release [18,19]. PHBV is a naturally occurring biodegradable polyester with characteristic surface erosion [20], making it a suitable candidate for biodegradable sensors [21]. PHBV has been reported to be completely biodegradable with >80% degradation within 80 days [22] and potentially improve plant growth [23]. PHBV will act as a printing substrate for the conductive part in the sensor. Beeswax and soy wax are natural biodegradable substances with no known detrimental effects on the environment or plant growth. In a study by McDonough et al. [22], more than 60% degradation occurred within 28 days in an OECD 301B Ready Biodegradability Test. In another study by Hatzinger and Alexander [24], beeswax showed a linear trend in mineralization, and >40% of beeswax was degraded by 90 days, indicating that beeswax is not persistent in the environment. Soy wax can refer to either fully (FHSBO) or partially hydrogenated soybean oil (PHSBO). Stearic acid, which comprises 80% of FHSBO [25,26,27], degrades 31% in 28 days in soil in non-accelerated conditions [28]. PHSBO, which is used in this study, consists of several long-chain fatty acids [26]. Microbes that can degrade these fatty acids have been found in soil [29]. Blends of soy and beeswax have been used as stimulants for microbial growth in petroleum remediation [30]. Due to differential rates of degradation of the waxes, our approach of using a blend of beeswax and soy wax could be beneficial to avoid measurement drifts before crop maturity. Balsa (*Ochroma pyramidale*) is a tropical hardwood, and it is highly popular in the timber industry due to fast plant growth, lightweight wood, and good mechanical properties [31]. Balsa wood, structurally made from cellulose, hemicellulose, and lignin, decomposes rapidly in the presence of water [32]. No detrimental impact of balsa wood on plant growth and development and the environment has been studied or reported.

This study was conducted to assess (i) the degradation of biodegradable soil moisture sensor components in different soil media and (ii) the effect of materials and their degradation on maize growth and development.

## 2. Materials and Methods

### 2.1. Study Design and Sensor Component Materials

This study was conducted from March 2020 to July 2020 in the Plant Growth Facility (PGF) greenhouse located at Colorado State University, Fort Collins, Colorado, USA (40°34′018.0′′ N 105°04′52.3′′ W). The experiment was set up as a completely randomized design in three blocks of growing media including (i) silica sand, (ii) Promix potting soil mix (Miracle-Gro, Lawn Products Inc., OH, Marysville, USA), and (iii) field soil classified as fine-loamy, mixed, superactive, mesic Aridic Haplustalf [33] and collected at 0–15 cm depth from a maize field at Colorado State University, Agricultural Research Development and Education Center, located in Fort Collins, CO, USA (40°40′38.24′′ N, 104°58′44.76′′ W). Five candidate sensor component materials Table 1, selected from previous preliminary greenhouse and accelerated degradation studies, and a control (no materials) were tested in each growing media to assess the degradation rate and their effects on maize growth development. A summary of dimension of materials is presented in Table 2. The experimental layout of the study is shown in Table 3.

Plastic pots (15.24 cm diameter and 15.24 cm height) were filled with growing media (silica sand, Promix soil mix, and field soil). Three replicate sensor materials (experimental unit for objective 1) were placed at 10 cm soil depth and four maize seeds (DKC 46-20 hybrid) were planted in a square pattern at 2.5 cm depth in each pot.

### 2.2. Crop Management

At the time of planting, 2 g of granular 15:9:12 NPK (Osmocote Plus, Scotts Miracle-Gro, Marysville, OH, USA) fertilizer was applied in each plot. After plants emerged, 100 mL of 200 ppm nitrogen solution was supplied twice a week. The concentration of N solution was increased to 400 ppm at 30 days after planting (DAP) to accommodate higher nitrogen demand of plants. At 30 DAP, 0.02 g (equivalent to 11.2 kg ha^−1^) of iron (Fe) and 0.02 g (equivalent to 11.2 kg ha^−1^) of zinc (Zn) were supplied to the plants to avoid micronutrient deficiency. An automated drip irrigation setup was created to supply 50 mL water twice a day (at 08:00 and 15:00). After two weeks of emergence, plants were thinned to establish two healthy plants (experimental unit for objective 2) in each pot. The experiment was managed as a typical greenhouse-based study [34,35,36,37].

### 2.3. Plant and Material Data Collection

Plants and materials from pots were retrieved according to the harvest schedule by growth stages as shown in the experimental layout Table 3, where H1 corresponds to harvest 1 at 30 DAP, H2 corresponds to harvest 2 at 60 DAP, H3 corresponds to harvest 3 at 90 DAP, and H4 corresponds to harvest 4 at 120 DAP. C1, C2, and C3, correspond to control plots (without materials) in each growing medium. Control pots, C1, C2, and C3 were harvested with H2, H3, and H4 harvests, respectively. At H1, no control pots were harvested because plants were very young or still germinating in soil pots. Each plant from the harvested pots was weighed immediately to record green biomass. In addition, height of each plant was measured using a measuring tape at every harvest. Plants were air-dried for 14 days and were weighed again to obtain dry biomass. When the plant biomass was constant for two days in a row, it was considered as dry biomass. For the sake of consistency, all samples were air-dried for 14 days. Materials were measured in terms of length, width, thickness, and weight before being placed in the pots. Materials were retrieved from harvested pots, washed with deionized water to remove any visible debris, and air-dried for seven days before making measurements. Length, width, and thickness of each material were measured using a 4-point precision vernier caliper (Stahlwille Co., Wuppertal, Germany), and weight was measured using a 4-point precision digital balance (Metler Toledo LLC, Columbus, OH, USA). Each material had 36 replicates, and every replicate was measured three times and an average value was obtained for each material attribute. The mean and standard deviation (SD) presented in Table 2 represent all (36) replicate materials of same kind. Percentage change in weight and dimension of materials at each growth stage was obtained by using the following formula.

$$\%\;change\;in\;attribute=\frac{Initial\;Value-Harvest\;Value}{Initial\;Value}\;\times \;100$$

### 2.4. Statistical Analysis

At each growth stage (30, 60, 90, and 120 DAP), for each growing media, plant attributes (plant height, green biomass, and dry biomass) were analyzed using ANOVA to detect significant differences between control plants and plants from material treatments (α = 0.05). One-factor ANOVA model with materials as the independent factor was used to model every plant attribute as shown in Equation (1).

(1) $$Y_{ij}=\mu +\tau_{i}+\varepsilon_{ij}$$

where $Y_{ij}$ is the value of *j*th subject in treatment group *i*, *µ* is the grand mean, $\tau_{i}$ is the treatment effect, and $\varepsilon_{ij}$ is the error term associated with $Y_{ij}$.

Tukey’s HSD (Honestly Significant Difference) [38] test was used to obtain and compare the pairwise difference between treatments. Material degradation between growth stages (30, 60, 90, and 120 DAP) was also compared for each material and growing media using ANOVA. The one-way ANOVA model, as shown in Equation (1), with growth stage as the independent factor was utilized to model % change in each material dimension. Line plots were created to visualize rates of degradation of material with time. All statistical analyses were conducted using the R statistical software package [39].

## 3. Results

### 3.1. Degradability of Materials

**Figure 1:**
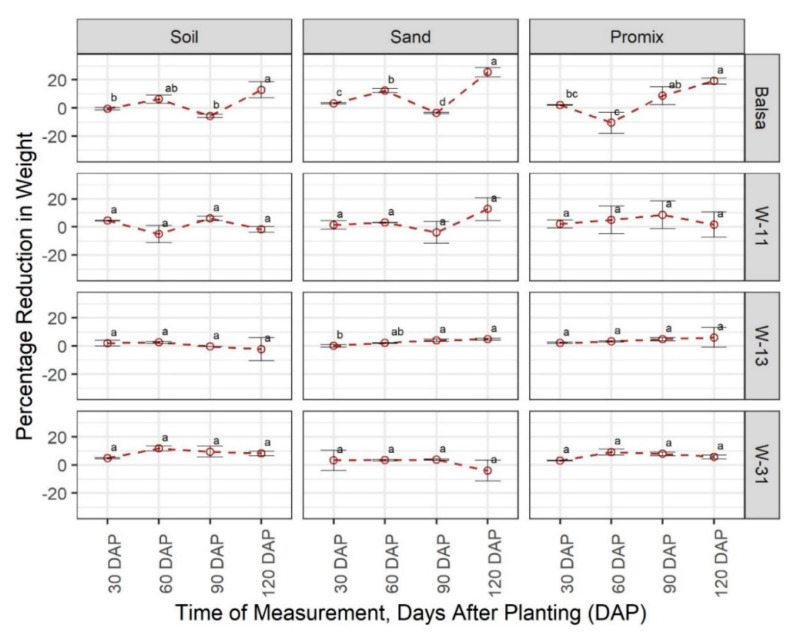
Comparison of the percentage change in material weight in three growing media (Promix, sand, and soil) at four growth stages (30, 60, 90, and 120 days after transplanting (DAP)). Different lowercase letters indicate significant differences in % weight change between different growth stages, at α = 0.05, within each growing media and material, whereas the same letters indicate no significant difference across compared treatments. W-11, 1:1 blend of beeswax and soy wax; W-13, 1:3 blend of beeswax and soy wax; and W-31, 3:1 blend of beeswax and soy wax.

All materials under consideration showed consistency in terms of weight, length, width, and thickness across all growing media and growth stages, except PHBV, which decomposed completely within 30 DAP in all growing media (Figure S2, in the supplementary material). In terms of material weight, balsa wood showed a general increase across all growing media and growth stages (Figure 1). All three wax blends showed considerable stability in terms of weight, with a range of −5% to 14%. The percentage change in weight in W-11, W-13, and W-31 wax blends did not show any significant difference (*p* = 0.05) across growth stages in any growth media, except W-11 in sand. Even in that particular instance, weight reduction was not observed. There was some variability when the weight change was compared between materials at each growth stage. Balsa wood exhibited the most variation at each growth stage due to unexpected weight gain (Figure 1).

**Figure 2:**
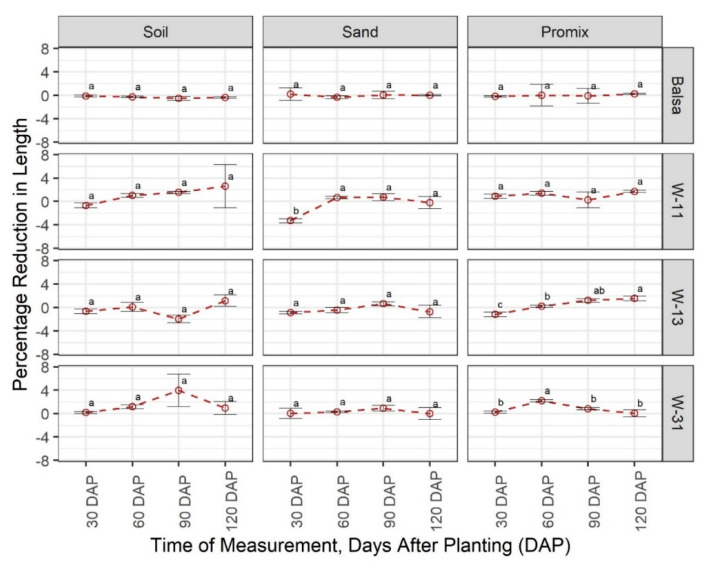
Comparison of percentage change in material length in three growing media (Promix, sand, and soil) at four growth stages (30, 60, 90, and 120 days after transplanting (DAP)). Different lowercase letters indicate significant differences in % length change between different growth stages, at α = 0.05, within each growing media and material, whereas same letters indicate no significant difference across compared treatments. W-11, 1:1 blend of beeswax and soy wax; W-13, 1:3 blend of beeswax and soy wax; and W-31, 3:1 blend of beeswax and soy wax.

**Figure 3:**
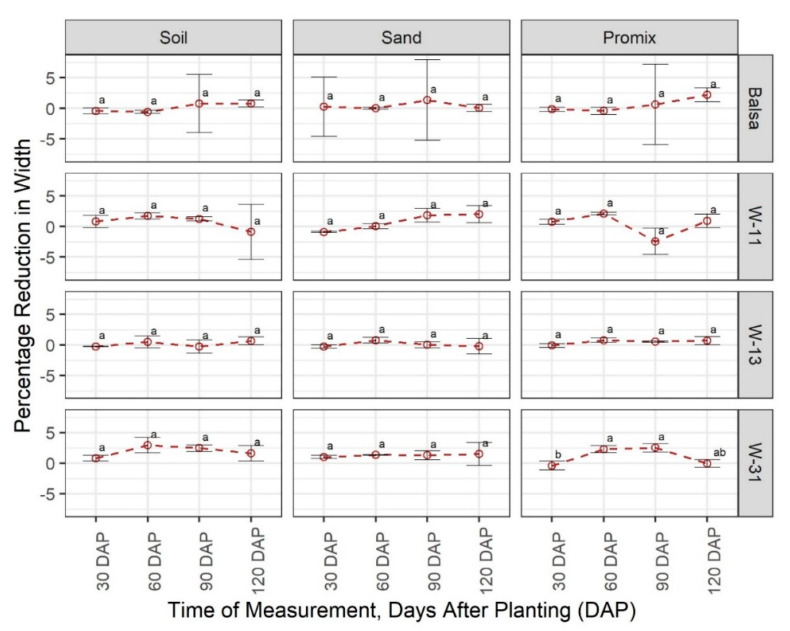
Comparison of percentage change in material width in three growing media (Promix, sand, and soil) at four growth stages (30, 60, 90, and 120 days after transplanting (DAP)). Different lowercase letters indicate significant differences in % width change between different growth stages, at α = 0.05, within each growing media and material, whereas same letters indicate no significant difference across compared treatments. W-11, 1:1 blend of beeswax and soy wax; W-13, 1:3 blend of beeswax and soy wax; and W-31, 3:1 blend of beeswax and soy wax.

In contrast to weight change, balsa wood was highly consistent in terms of length change (average −0.12%) across growth media and growth stages with no statistical difference at α = 0.05 (Figure 2). Three wax blends were also consistent in terms of percentage length change (average 0%), except W-11 at 30 DAP, W-13 at 30 DAP, and W-31 at 60 DAP. As expected, change in material width (Figure 3) exhibited a similar response as the changes in length. The W-13 blend in Promix at 60 and 90 DAP showed an increase in width, which could be attributed to mold buildup as observed in the study (Figure S3, in the supplementary material) and measurement errors.

**Figure 4:**
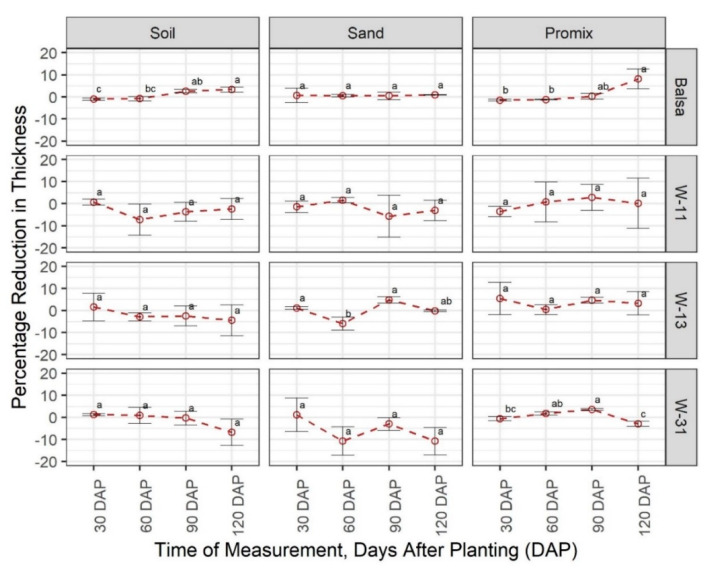
Comparison of percentage change in material thickness in three growing media (Promix, sand, and soil) at four growth stages (30, 60, 90, and 120 days after transplanting (DAP)). Different lowercase letters indicate significant differences in % thickness change between different growth stages, at α = 0.05, within each growing media and material, whereas same letters indicate no significant difference across compared treatments. W-11, 1:1 blend of beeswax and soy wax; W-13, 1:3 blend of beeswax and soy wax; and W-31, 3:1 blend of beeswax and soy wax.

As was observed for the changes in the weight, balsa wood showed a general increase in thickness (Figure 4) across all growth media and growth stages. Material thickness is an important attribute for an encapsulant, and consistency in the thickness of all wax blends was observed in most cases (Figure 4). The W-11 blend was the most consistent wax blend with no significant differences between any growth stages in all growth media. W-13 was consistent in most of the cases except at 60 DAP in the sand, whereas the W-31 blend showed a significant reduction in thickness at 120 DAP in Promix.

### 3.2. Effect of Materials on Growth and Development of Plants

**Figure 5:**
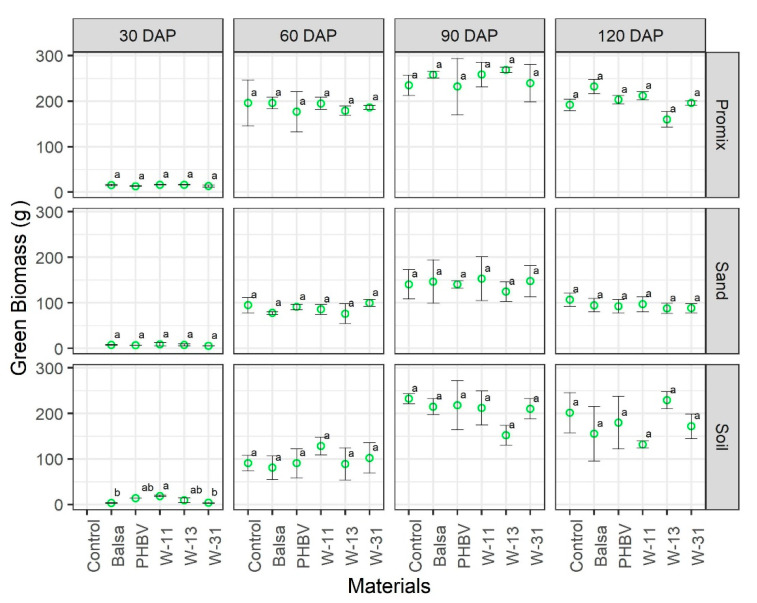
Comparison of maize green biomass (g) in three growing media (Promix, sand, and soil) and four growth stages (30, 60, 90, and 120 days after transplanting (DAP)). Different lowercase letters indicate significant differences at α = 0.05, whereas same letters indicate no significant difference across compared treatments.

**Figure 6:**
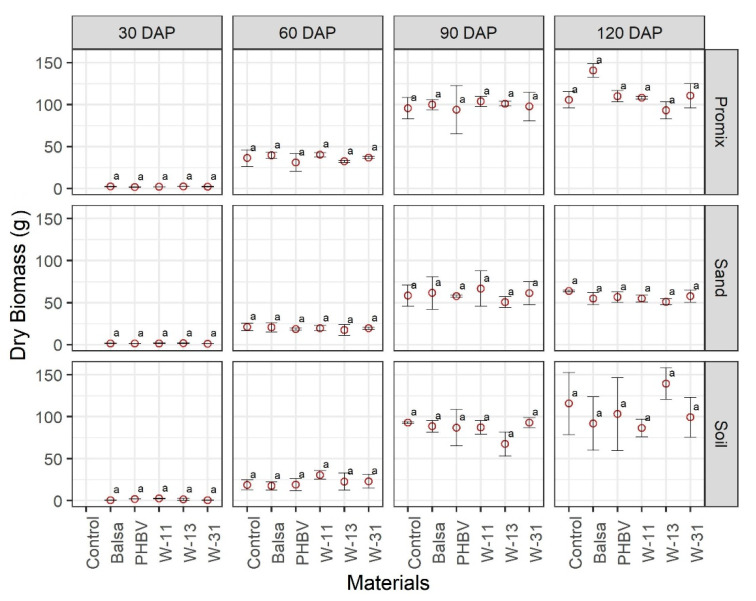
Comparison of maize dry biomass (g) in three growing media (Promix, sand, and soil) and four growth stages (30, 60, 90, and 120 days after transplanting (DAP)). Different lowercase letters indicate significant differences at α = 0.05, whereas same letters indicate no significant difference across compared treatments.

**Figure 7:**
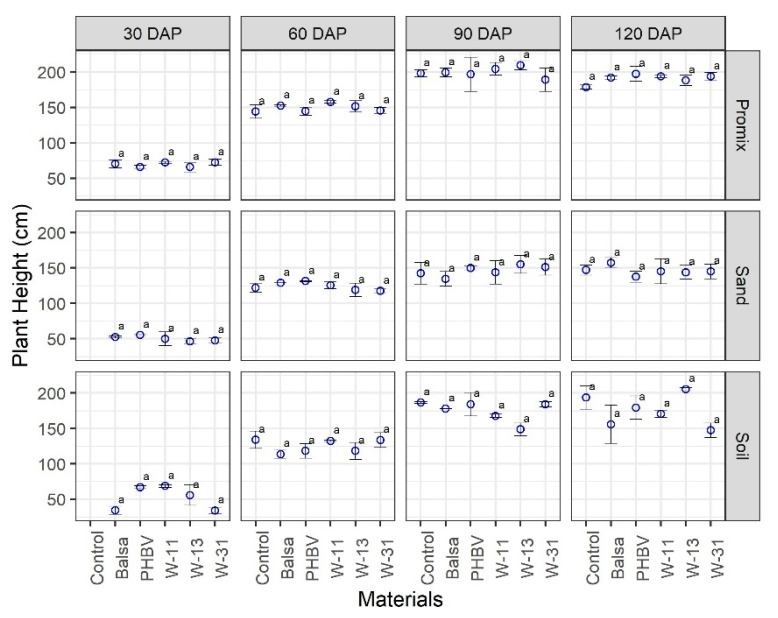
Comparison of maize plant height (cm) in three growing media (Promix, sand, and soil) and four growth stages (30, 60, 90, and 120 days after transplanting (DAP)). Different lowercase letters indicate significant differences at α = 0.05, whereas same letters indicate no significant difference across compared treatments.

Dry plant biomass was affected by growing media as expected, and dry weight was greater in soil and Promix growing media as compared with sand at all growth stages. The materials did not exhibit a significant effect on the growth and development of maize plants across three growing media and four growth stages (Figure 5, Figure 6 and Figure 7). Green biomass was not affected by any of the materials in any of the growing media except soil at 30 DAP, where green biomass was significantly different among treatments (Figure 5). Some soil pots had to be reseeded because of delayed/no germination caused by soil compaction and waterlogging, following irrigation, which might be attributed to significant differences in green biomass in soil pots at 30 DAP. Similarly, there was no significant effect of any materials on dry biomass as compared to the control pots, in any growing medium at any growth stage (Figure 6). Likewise, the materials and their degradation did not have any significant effect on plant height at any growth stage or growth medium (Figure 7).

## 4. Discussion

Screening and pre-assessment of suitable biodegradable materials are crucial steps for developing biodegradable soil moisture sensors. Not only should the sensor last an entire crop growing season, but the materials used should not have any detrimental impact on crop growth and development, or soil and environmental health. The candidate materials in this study were selected based on a rigorous literature review and a preliminary lab study. The structural substrate (balsa wood) is a base, on top of which a printing substrate (PHBV) will be attached. A metal electrode will be printed on top of the printing substrate, and the structural setup will be covered with an encapsulant (wax blends). The sensor will fail as soon as the encapsulant degrades, and water flows in.

PHBV is widely used in other fields, and its degradability is well understood. Highly malleable mechanical property and no known biotoxicity of PHBV make it an excellent candidate as a printing substrate. Consistent with a previous study [22], PHBV was degraded within 30 days in soil and Promix medium and within 60 days in sand medium (Figure S2, in the supplementary material). Swift degradation of PHBV is desirable to allow rapid detection of failed sensors.

Balsa is a natural, biodegradable, fast-growing, lightweight wood with excellent mechanical properties [31], which makes it a suitable candidate for a short-term biodegradable sensor. In theory, balsa wood would come in contact with soil water only when the encapsulant degrades; thus, the mechanical property is the most important parameter for balsa wood as compared to degradability. In terms of degradation, balsa wood did not have a notable reduction in weight, length, width, and thickness up to 120 days in any growth medium. In most instances (8 out of 12), balsa wood recorded an increase in weight (Figure 1), which might be attributed to mold and soil particles attached to the wood at the time of removal and residual moisture after drying. In the soil growth medium at 90 DAP, two balsa woods were broken due to soil compaction and the force of extracting the materials (Figure S4, in the supplementary material); however, in 34 instances out of 36, balsa wood was intact without notable degradation. The durability of balsa wood strengthens the possibility of using balsa wood as the structural substrate in soil moisture sensors.

In terms of degradation, the encapsulant is the most important component of the sensor because as soon as the water goes through the encapsulant, the sensor will fail rapidly. Beeswax and soy wax were selected as encapsulants due to their hydrophobic nature and slow degradation rate. Based on the results of a preliminary study (data not shown), a blend of these two waxes rather than one of them was superior in terms of maneuverability and erodibility. All wax blends were consistent in terms of weight, length, width, and thickness throughout the study period (Figure 1, Figure 2, Figure 3 and Figure 4). However, surface cracks were visible at 120 DAP in W-11 and W-13 blends (Figure S4, in the supplementary material). Surface cracks could sip water in, causing sensor failure; thus, W-31 blend is the recommended encapsulant for the sensors. The degradation rate of wax in this study was slower as compared with the rates reported in the literature [25,26,27], which could be attributed to the ratio of surface area and mass. The wax blends in this study were cube-shaped with a low surface-area-to-mass ratio; however, dimensions of waxes were not reported in the abovementioned studies.

The materials under consideration do not have any reported environmental issues upon degradation; however, their impact on crop growth and health have not been studied before. The materials under study did not have any detrimental effect on maize growth and development (Figure 5, Figure 6 and Figure 7 and Figure S5). Results from this study are of tremendous significance because the pots used in this study were 15 cm in diameter and 15 cm in height, supporting two maize plants. The rate of sensor deployment in an actual maize field is approximately 8–10 sensors per hectare; thus, we can be confident that sensor materials and their degradation do not pose any significant threat to crop growth, health, and yield.

## 5. Conclusions

Biodegradable soil moisture sensors could be the next big thing in perfecting precision agriculture. Results from this study showed that all candidate sensor components (structural substrate (balsa wood), printing substrate (PHBV), and encapsulants (wax blends)) had the desirable degradability attributes for creating biodegradable soil sensors, and their degradation did not have any detrimental impacts on maize crop growth and development. Further studies are required to fully understand the degradability and functionality of biodegradable sensors using a finished sensor setup.

## Figures and Tables

**Table 1:** Description of candidate sensor materials used in the study.

Material	Abbreviation	Function in Sensor	Description
Balsa wood	B	Structural substrate	Lightweight natural substance
Poly(3-hydroxybutyrate-co-3-hydroxyvalerate)	PHBV	Printing substrate	Rapidly biodegradable carbon polymer
1:1 Beeswax:soy wax blend	W-1:1	Encapsulant	Beeswax, natural wax produced by bees. Denser than soy wax
1:3 Beeswax:soy wax blend	W-1:3	Encapsulant	Soy wax, natural wax produced from soybean. Softer than beeswax
3:1 Beeswax:soy wax blend	W-3:1	Encapsulant	

Soy wax, Golden Brands Natural Soy Wax (444), Lone Star Candle Supply Inc., Keller, TX, USA. Beeswax, refined beeswax, Sigma-Aldrich, MO, St. Louis, USA. PHBV, Goodfellow Corporation, Coraopolis, PA, USA. Balsa wood, Revell, Home Depot, CO, Denver, USA.

**Table 2:** Mean and standard deviation of material attributes (length, width, thickness, and weight) before placement in the pots.

Material	Attribute	Mean	SD
W-11	Length (mm)	14.991	0.092
	Width (mm)	15.021	0.124
	Thickness (mm)	5.238	0.531
	Weight (g)	1.068	0.109
W-13	Length (mm)	14.977	0.099
	Width (mm)	14.967	0.104
	Thickness (mm)	5.473	0.499
	Weight (g)	1.081	0.097
W-31	Length (mm)	15.181	0.141
	Width (mm)	15.171	0.082
	Thickness (mm)	5.118	0.661
	Weight (g)	1.066	0.130
Balsa	Length (mm)	50.525	0.627
	Width (mm)	24.963	1.101
	Thickness (mm)	6.530	0.123
	Weight (g)	1.174	0.162

W-11, 1:1 blend of beeswax and soy wax; W-13, 1:3 blend of beeswax and soy wax; and W-31, 3:1 blend of beeswax and soy wax. SD, standard deviation.

**Table 3:** Experimental layout showing blocks and location of pots.

Soil				Promix				Sand			
W-31-H1	B-H1	W-11-H3	W-11-H4	W-31-H2	W-11-H2	W-13-H2	B-H1		W-11-H1	W-13-H1	C1
C2	W-31-H2	B-H3	PHBV-H3	W-31-H4	W-31-H1	C2	W-11-H4	B-H4	PHBV-H1	PHBV-H3	W-31-H3
W-13-H2	W-13-H1	W-31-H3	PHBV-H2	W-31-H3	PHBV-H1	PHBV-H3	PHBV-H4	B-H2	W-11-H4	W-11-H2	C2
W-31-H4	C1	W-13-H4	B-H4	B-H2		W-13-H4	B-H4	C3	W-13-H3	W-31-H1	W-11-H3
W-11-H2	C3	PHBV-H4		W-11-H1	W-11-H3	W-13-H3	C3	W-13-H4	B-H3	W-13-H2	P HBV-H2
PHBV-H1	B-H2	W-13-H3	W-11-H1	C1	PHBV-H2	W-13-H1	B-H3	W-31-H4	B-H1	W-31-H2	PHBV-H4

Soil, Promix, and sand columns indicate the three growing media. B, balsa wood; W, wax blend, the number following “W” represents the ratio of beeswax and soy wax in the blend; PHBV, poly(3-hydroxybutyrate-co-3-hydroxyvalerate); C, control pots; H, harvest, the numbers following “H” represent the harvest time (H1: 30 days after planting (DAP), H2: 60 DAP, H3: 90 DAP, and H4: 120 DAP). The location of pots is adjusted here for the ease of visualization. Empty cells had a pot and plants same as control pots.

## Supplementary Materials

The following are available online at https://www.mdpi.com/1424-8220/20/21/6154/s1, Figure S1: Pictures showing (a) balsa wood, and three beeswax:soy wax blends; (b) 1:1, (c) 1:3, and (d) 3:1 before placing them in the pots, Figure S2: Poly(3-hydroxybutyrate-co-3-hydroxyvalerate) (PHBV) decomposition in sand at 30 DAP, Figure S3: Pictures showing material degradation at 30 days after planting (DAP), denoted by H1 and 60 DAP, denoted by H2, Figure S4: Pictures showing material degradation at 90 days after planting (DAP), denoted by H3 and 120 DAP, denoted by H4, Figure S5: Growth of maize plants in three growing media at four growth stages. DAP, days after planting.
