# Supplementary material for: Degradability of Biodegradable Soil Moisture Sensor Components and Their Effect on Maize (Zea mays L.) Growth
Source: Sensors (Basel). 2020 Oct 29;20(21):6154. doi: 10.3390/s20216154 (PMC7663592; doi:10.3390/s20216154)
Supplement: Supplementary file 1 [file sensors-20-06154-s001.pdf]

Supplementary Material

Degradability of Biodegradable Soil Moisture Sensor Components and their Effect on Maize (*Zea Mays* L.) Growth

Subash Dahal [1], Wubengeda Yilma [1], Yongkun Sui [2], Madhur Bharat Atreya [2], Samantha Bryan[1], Valerie Davis[1], Gregory Lewis Whiting [2] and Raj Khosla [1*]

[1] Department of Soil and Crop Sciences, Colorado State University, Fort Collins, CO 80523-1170, USA; subash.dahal@colostate.edu ; wub.yilma@colostate.edu; samisam@rams.colostate.edu ; valerie.davis@colostate.edu ; raj.khosla@colostate.edu

[2] Paul M. Rady Department of Mechanical Engineering, University of Colorado Boulder, CO 80309-0427, USA ; madhur.atreya@colorado.edu ; yongkun.sui@colorado.edu ; gregory.whiting@colorado.edu

* Correspondence: raj.khosla@colostate.edu; Tel.: +1-970-491-1920

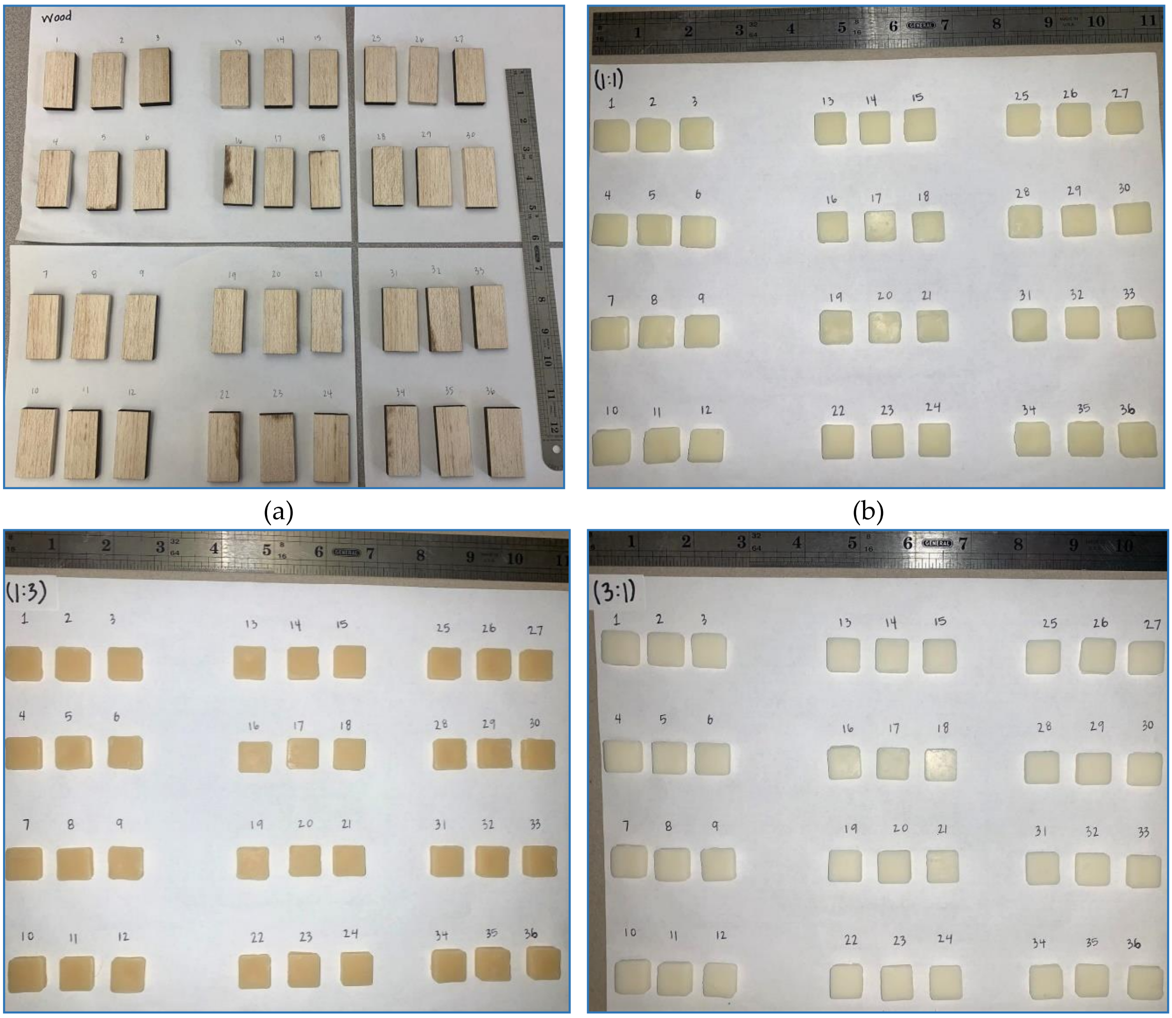

(a) (b) (c) (d)

Figure S1. Pictures showing (a) balsa wood, and three beeswax:soy wax blends; (b) 1:1, (c) 1:3, and (d) 3:1 before placing them in the pots.

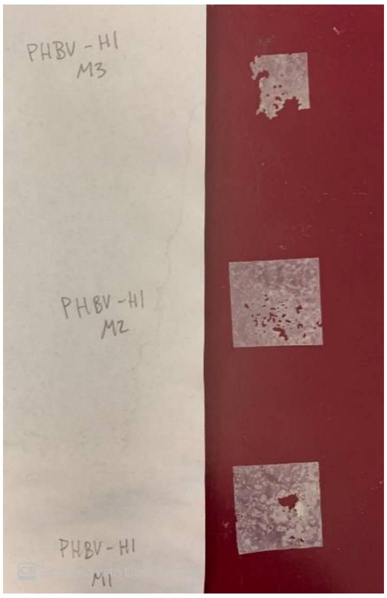

Figure S2. Poly(3-hydroxybutyrate-co-3-hydroxyvalerate) (PHBV) decomposition in sand at 30 DAP.

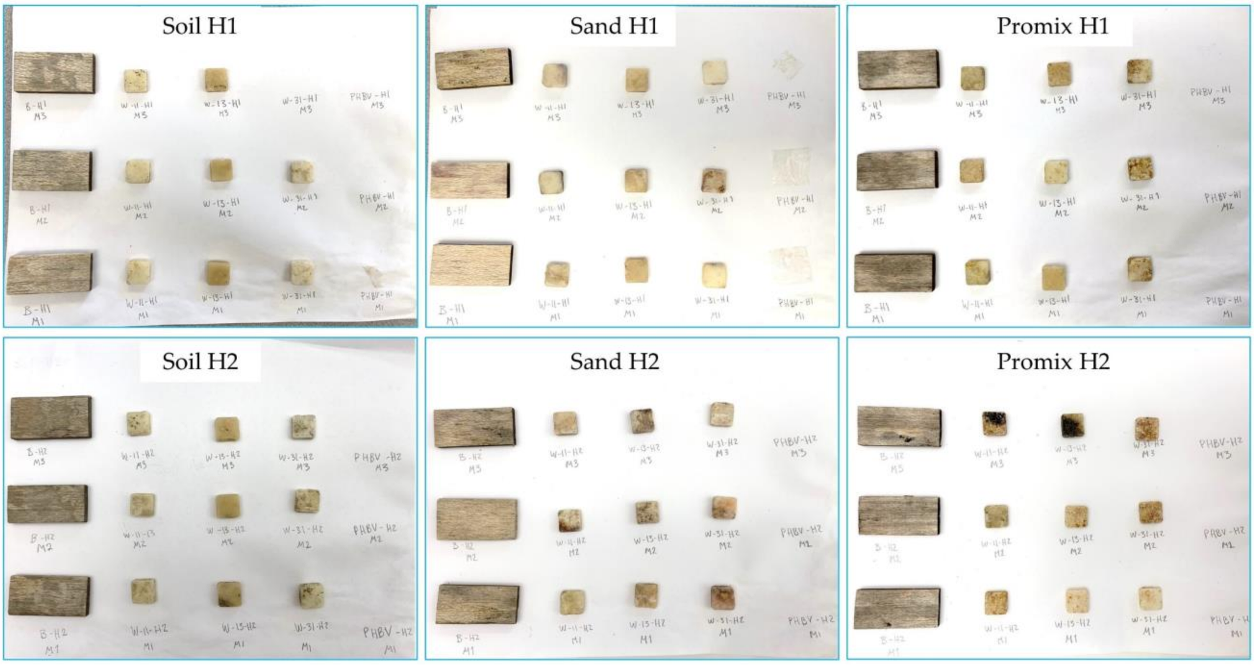

Figure S3. Pictures showing material degradation at 30 days after planting (DAP), denoted by H1 and 60 DAP, denoted by H2.

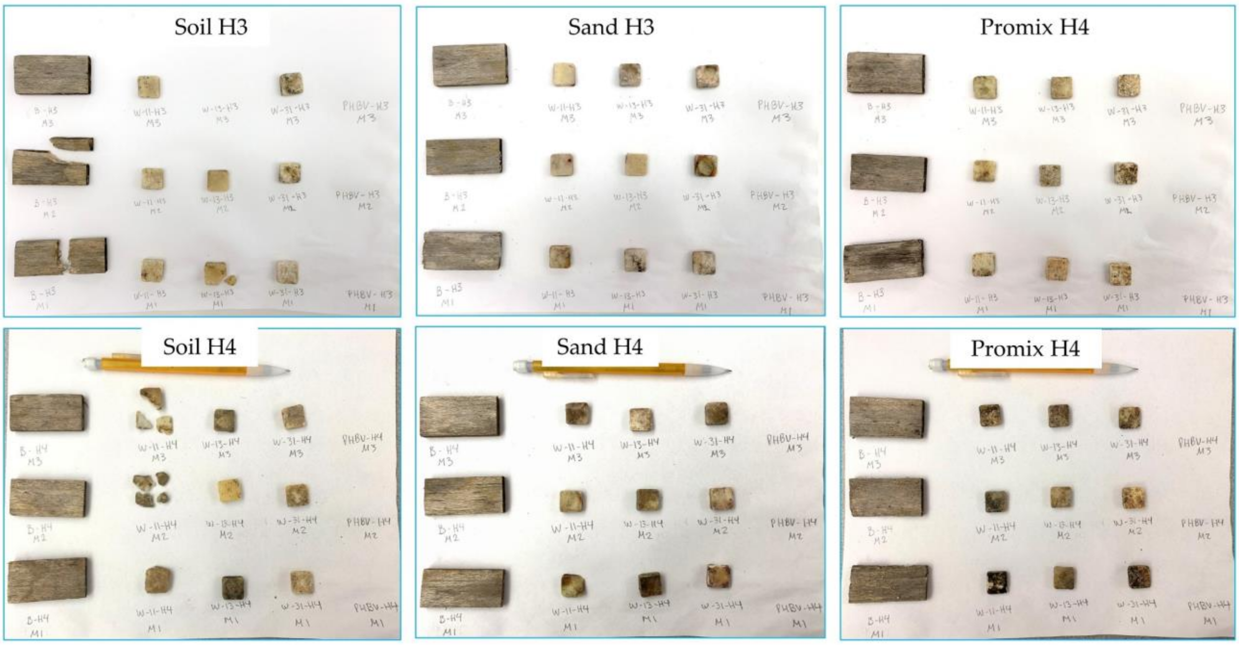

Figure S4. Pictures showing material degradation at 90 days after planting (DAP), denoted by H3 and 120 DAP, denoted by H4.

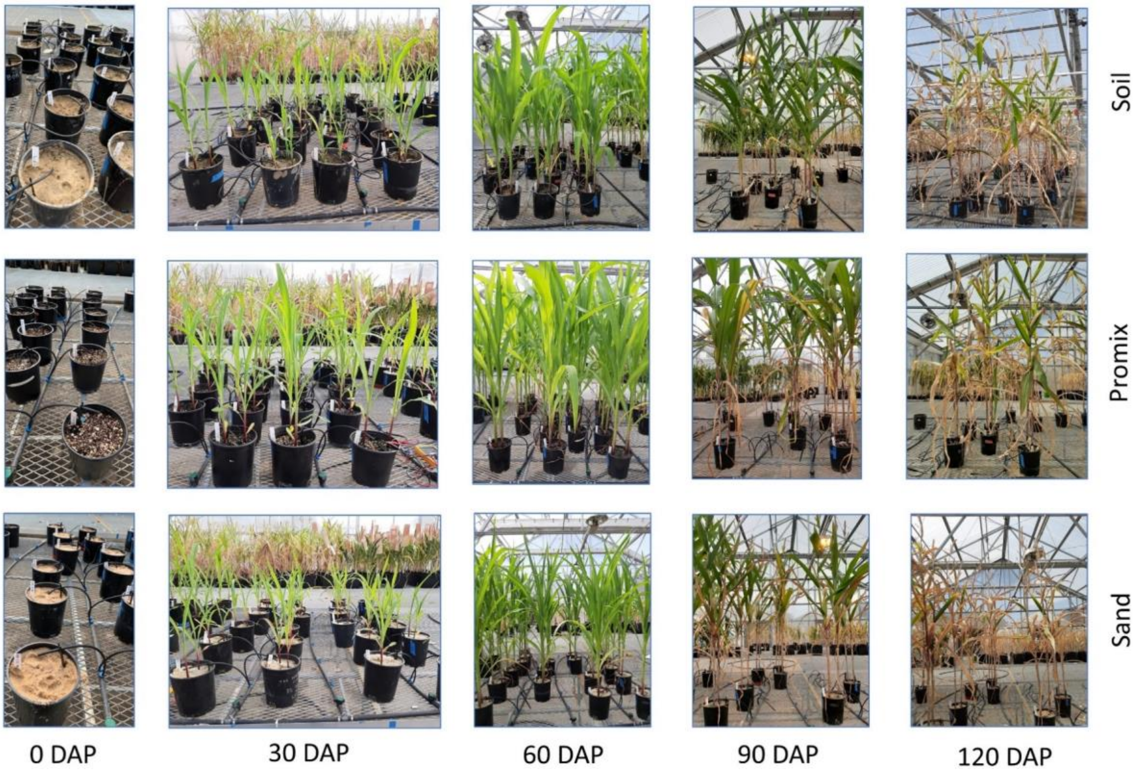

Figure S5. Growth of maize plants in three growing media at four growth stages. DAP, days after planting.
